# Supplementary material for: Loops around the Heme Pocket Have a Critical Role in the Function and Stability of BsDyP from Bacillus subtilis
Source: Int J Mol Sci. 2021 Oct 8;22(19):10862. doi: 10.3390/ijms221910862 (PMC8509576; doi:10.3390/ijms221910862)
Supplement: Supplementary file 1 [file ijms-22-10862-s001.zip › ijms-1404479-supplementary.pdf]

Supplementary Information

Loops around the heme pocket have a critical role in function and stability of *Bs*DyP from *Bacillus subtilis*

Carolina F. Rodrigues[1], Patrícia T. Borges[1], Magali F. Scocozza[2], Diogo Silva[1], André Taborda[1], Vânia Brissos[1], Carlos Frazão[1], Lígia O. Martins[1]

Table S1. Specific activity and genotype of the best variants obtained in the first and second rounds of directed evolution. Synonymous substitutions are shown between brackets.

Generation	Variants	Specific activity (mU/mg)	Genotype
	WT	4.9 ± 0.6	-
1st G	1F9	23.2 ± 1.5	S325P (L223, K227)
	3G5	17.3 ± 4.2	A330V L166Q V284A T296S
2nd G	50F7	57.5 ± 4.7	S325P K220R (L223 K227 S289 V316 L348)
	54D6	46.4 ± 2.1	S325P K317E (L223 K227)
	51E9	44.3 ± 5.7	S325P K248E (L223 K227)
	56C8	19.3 ± 2.4	S325P E346V (L223 K227 L166)

Table S2. Genotype of variants generated in the third round of evolution using DNA-shuffling. Relative activity for DMP (in relation to 50F7) and sequence analysis of the 25 most active variants after shuffling the genes of four best variants from the second generation, <u>50F7</u> (S325P, K220R, and silent L223L, K227K, S289S, V316V, L348L), <u>51E9</u> (S325P, K248E, and silent L223L, K227K), <u>54D6</u> (S235P, K317E, and silent L223L, K227K) and <u>56C8</u> (S235P, E346V, and silent L223L, K227K, L166L) and <u>3G5</u> (A330V, L166Q, V284A,T296S) from the first generation. Synonymous mutations are between brackets.

Variant	5G5	7E7	3C7	6B8	2C5	7E10	5D6	2E4	11B9	6F10	5E6	5C9	5E10	6C10	5C8	5E8	5F5	10B6	6B10	6G8	10D5	6G10	5G8	3C8	7B8
Relative Activity AVG	2.5	1.9	2.0	2.1	1.9	1.7	1.6	1.2	1.6	1.6	1.1	1.5	1.4	1	1.3	1.1	1.0	1.0	1.3	1.1	1.2	1.0	0.9	0.8	0.8
STD	0.5	0.3	0.2	0.2	0.4	0.3	0.3	0.3	0.2	0.4	0.2	0.2	0.4	0.5	0.2	0.4	0.3	0.2	0.7	0.5	0.3	0.4	0.4	0.2	0.3
S325P	×	×	×	×	×	×	×	×	×	×	×	×	×	×	×	×	×	×	×	×	×	×	×	×	×
A330V	×	×	×	×	×	×				×	×														
L166Q																									
V284A									×	×	×									×	×	×			×
T296S																							×	×	×
K220R							×	×				×	×	×											
K248E							×	×	×										×			×	×	×	
K317E	×	×			×	×			×										×	×				×	
E346V			×	×	×	×									×	×	×	×	×	×					
(L166L)								×			×			×	×								×	×	
(L223L)	×	×	×	×	×	×	×	×	×	×	×	×	×	×	×	×	×	×	×	×	×	×	×	×	×
(K227K)	×	×	×	×	×	×	×	×	×	×	×	×	×	×	×	×	×	×	×	×	×	×	×	×	×
(S289S)	×			×				×	×	×									×	×				×	
(V316V)																	×								×
(L348L)									×	×		×													

Table S3. Specific activity of top variants obtained in the third round of evolution. Activity was measured in crude extracts after growth at a 50 mL-scale in 100 mM sodium acetate buffer, pH 3.8, 1 mM of DMP, and 0.2 mM H_2O_2 at room temperature. The progress of reaction was monitored by the increase of absorbance at 468 nm.

**Variants**	**Specific Activity (mU/mg)**
5G5	122.1 ± 8.4
7E7	71.9 ± 6.9
6B8	61.8 ± 5.3
3C7	23.3 ± 3.2
2C5	108.1 ± 4.7
7E10	69.3 ± 4.9

Table S4. Amino acid residues that limit the molecular tunnels in *Bs*DyP wild-type and 5G5 variant. The marked residues, D240 and R339, are the distal catalytic residues. The marked H326 is the proximal heme ligand. The symbol * represents the mutation A330V in 5G5 variant.

Enzyme	Chain	Residues	Radius (Å)	Length (Å)
Wild-type	**A**	H73, L223, P232, N234, **D240,** G241, N244, I278, F297, **H326**, L338, **R339,** R340, A341, F342, L359, F361, I362, S363, L379, L385	1.35	9.26
	**B**	H73, L223, N234, **D240,** G241, N244, I278, F297, **H326**, L338, **R339,** R340, A341, F342, L359, F361, I362, S363, L379, L385	1.38	9.17
5G5	**A**	H73, L223, T231, P232, N234, **D240,** G241, T242, G243, N244, I278, F297, **H326,** V327, S328, V330*, L338, **R339**, R340, A341, F342, L359, L360, F361, I362, M375, L379, L385	1.50	9.89
	**B**	H73, L223, T231, P232, N234, **D240**, G241, T242, N244, I278, F297, **H326,** V330*, L338, **R339,** R340, A341, F342, L359, L360, F361, I362, L379, L385	1.53	10.08
	**C**	H73, L223, T231, P232, N234, **D240,** G241, T242, G243, N244, I278, F297, **H326,** V327, S328, V330*, L338, **R339**, R340, A341, F342, L359, L360, F361, I362, M375, L379, L385	1.47	9.75
	**D**	H73, L223, T231, P232, N234, **D240**, G241, T242, N244, I278, F297, **H326,** L338, **R339,** R340, A341, F342, L359, L360, F361, I362, L379, L385	1.51	9.74

Table S5. Amino acid residues that limit the cavity in *Bs*DyP wild-type and 5G5 variant. The marked residues R339 is the distal arginine catalytic residue and H326 residue is the proximal heme ligand. The symbol * represents the mutation A330V in 5G5 variant.

**Enzyme**	**Chain**	**Residues**	**Volume (Å^3)**	**Area (Å^2)**	**Depth (Å)**
Wild-type	**A**	F238, K239, T242, G243, Q245, R299, G304, V316, **H326,** V327, A330, K331, G334, K335, Q336, I337, **R339**	187.14	278.03	11.63
	**B**	F238, K239, T242, G243, Q245, R299, G304, V316, **H326,** V327, A330, K331, G334, K335, Q336, I337, **R339**	179.25	258.75	11.65
5G5	**A**	F238, K239, T242, G243, Q245, F297, R299, R300, K301, G304, **H326,** V327, V330*, K331, G334, K335, Q336, I337, **R339**	274.02	347.43	14.43
	**B**	F238, K239, T242, G243, Q245, F297, R299, R300, S302, G304, P306, **H326,** V327, V330*, K331, G334, K335, Q336, I337, **R339**	283.45	330.24	11.19
	**C**	F238, K239, T242, G243, Q245,F297, R299, R300, K301, G304, P306, **H326,** V327, V330*, K331, G334, K335, Q336, I337, **R339**	283.59	353.32	11.00
	**D**	K239, T242, G243, Q245, R299, R300, K301, P306, P315, V316, **H326,** V327, V330*, K331, G334, K335, Q336, I337, **R339**	203.27	331.48	7.87

Table S6. Accessible surface area (ASA) values (%) of amino acid residues limiting the cavities in *Bs*DyP wild-type and 5G5 variant.

	ASA (%)					
Residues	***Bs*DyP WT**		***Bs*DyP 5G5**			
	**A**	**B**	**A**	**B**	**C**	**D**
F238	2	2	2	2	2	2
K239	12	12	25	26	25	13
T242	21	19	60	61	61	21
G243	23	24	13	21	22	20
Q245	36	33	34	34	35	34
F297	0	0	0	0	0	0
R299	5	5	18	18	19	5
R300	36	40	38	36	38	41
K301	8	8	9	8	9	7
S302	75	75	70	65	70	74
G304	5	4	36	33	34	5
P306	0	0	67	67	67	2
P315	63	68	-	-	-	70
V316	15	15	-	-	-	39
S/P325	0	0	34	32	32	10
H326	1	0	0	0	0	0
V327	0	0	1	1	1	0
A/V330	0	0	1	1	1	1
K331	40	40	52	50	51	45
G334	71	72	71	70	70	71
K335	30	32	30	31	32	30
Q336	36	37	31	40	39	40
I337	4	4	2	4	5	4
R339	6	8	6	6	6	6

Table S7. Aromatic residues composition in DyPs from different classes.

Class	Enzyme origin	PDB	Tryptophan residues (%)	Tyrosine residues (%)	Total residues
**P**	*Enterobacter lignolyticus*	5VJ0	4 (1.3)	9 (3.0)	298
	Klebsiella pneumoniae	6FKS	4 (1.3)	9 (3.0)	298
	Streptomyces lividans	6YRJ	2 (0.6)	5 (1.6)	316
	Vibrio cholerae	5DE0	2 (0.7)	9 (3.0)	305
**I**	***Bacillus subtilis***	**7PKX**	4 (1.1)	10 (2.8)	354
	Cellulomonas bogoriensis	6QZO	9 (2.5)	4 (1.1)	361
	Thermomonospora curvata	5JXU	7 (1.9)	3 (0.8)	360
	Streptomyces lividans	6GZW	6 (1.6)	3 (0.8)	377
**V**	*Amycolatopsis sp.*	4G2C	5 (1.1)	9 (1.9)	462
	Anabaena sp.	5C2I	8 (1.5)	13 (2.8)	468
	Auricularia auricula-judae	4AU9	4 (0.9)	7 (1.6)	448
	Bjerkandera adusta	3AFV	5 (1.1)	6 (1.4)	439

Table S8. Thermodynamic stability of *Bs*DyP wild-type and variant enzymes as monitored by fluorescence emission (tertiary structure) at 360 nm.

	**ΔG^{water} (Kcal·mol^{-1})**	**m (Kcal·mol^{-1}·M^{-1})**	**[GdnHCl]$_{50\%}$ (M)**
**WT**	7.0 ± 1.0	5.0 ± 1.0	1.37 ± 0.02
**1F9**	7.0 ± 2.3	5.9 ± 2.0	1.20 ± 0.01
**3G5**	3.9 ± 0.1	4.0 ± 0.3	0.96 ± 0.03
**54D6**	4.6 ± 0.3	4.9 ± 0.3	0.96 ± 0.01
**5G5**	5.0 ± 1.0	4.5 ± 0.4	1.1 ± 0.1

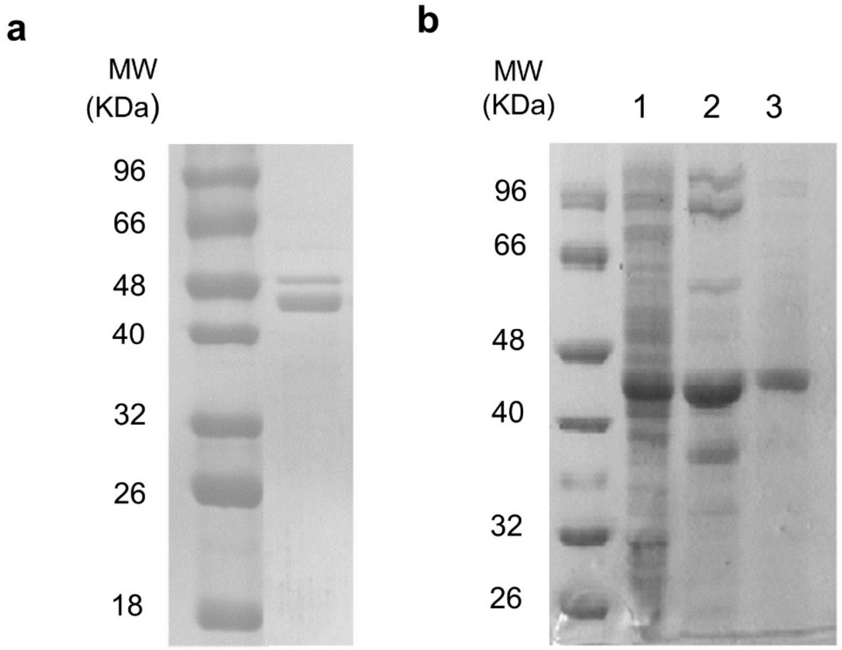

Figure S1. (a) SDS-PAGE analysis of purified preparations of *Bs*DyP with two bands: upper band is putatively assigned to a cytoplasmic enzyme containing the Tat signal peptide (MSDEQKKPEQIHRRDILKWGAMAGAAVAIGASGLGGLAPLVQTA) and the lower band to the mature, periplasmic enzyme without Tat signal peptide. **(b)** SDS-PAGE analysis of the purification of *Bs*DyP deleted in the signal peptide: crude extracts (1), after a SP-Sepharose (2) and Superdex-75 (3) chromatographic columns.

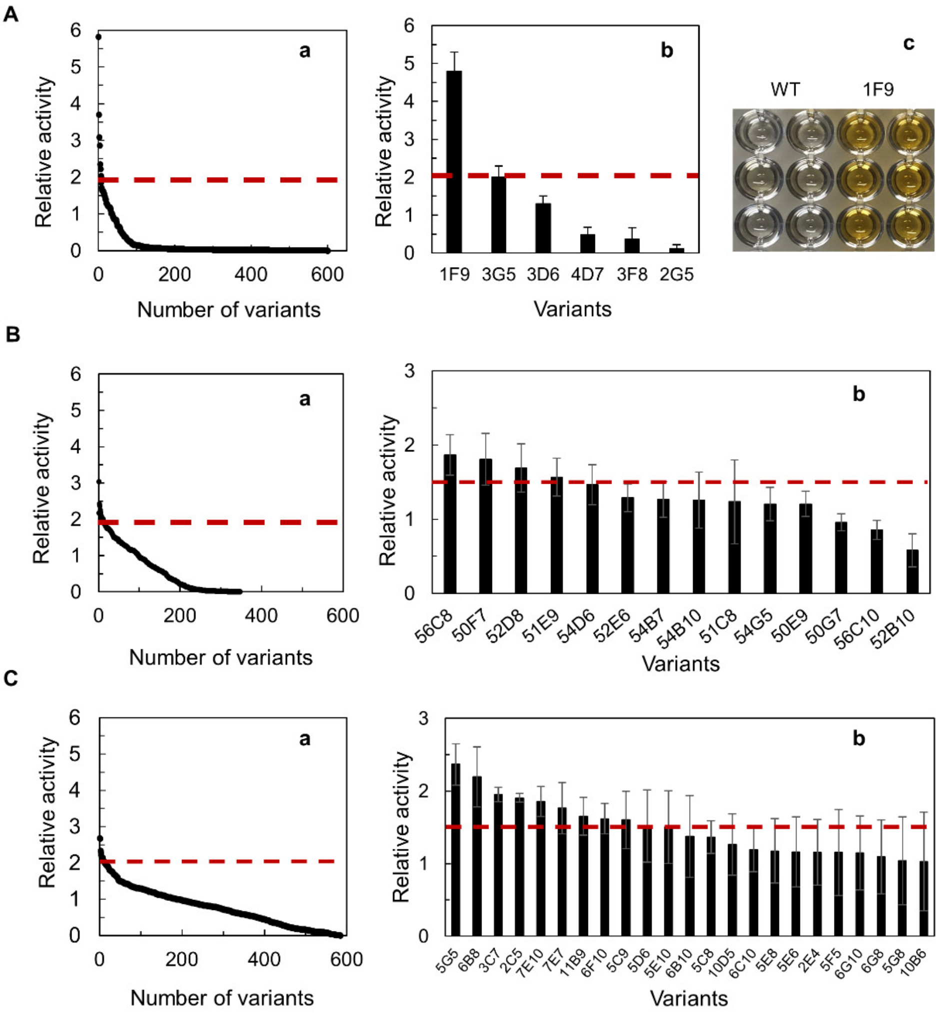

Figure S2. Screening of variants´ libraries during evolution. **(A)** Activity screening landscape of a total of 614 variants (*n*=1) (relative activity to wild-type) obtained in the first round of evolution **(a)**, re-screening of the six best variants (*n*=12) **(b)**, and a picture where it is visible the difference in activity between wild-type (left) and 1F9 (right) **(c)**. **(B)** Activity screening landscape of 365 variants (*n*=1) selected with activity from a total of 3500 clones from the second round of evolution screened in an "activity-on-plate" **(a)**, (relative activity to 1F9) and rescreening results of the 14 best variants (*n*=24) **(b)**. **(C)** landscape of the screening performed with 588 variants (*n*=1) selected with activity from a total of 3500 clones screened in a "activity-on-plate" from the third round of evolution **(a)** (relative to variant 50F7) and rescreening of the best 25 variants (*n*=24) **(b)**. In all figures the horizontal dashed line represents the threshold used to select the best variants.

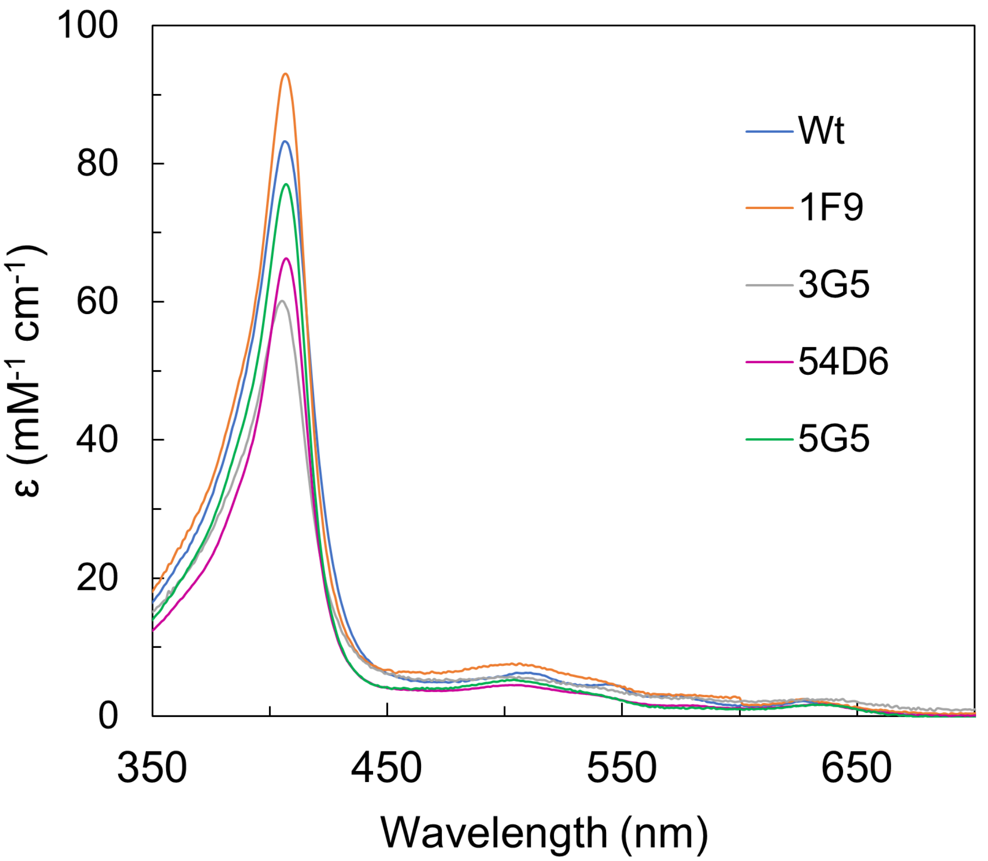

Figure S3. UV-vis spectra of purified *Bs*DyP wild-type and variants, with the Soret band at 406–407 nm, the Q bands at 502–505 nm and CT bands at 630 nm.

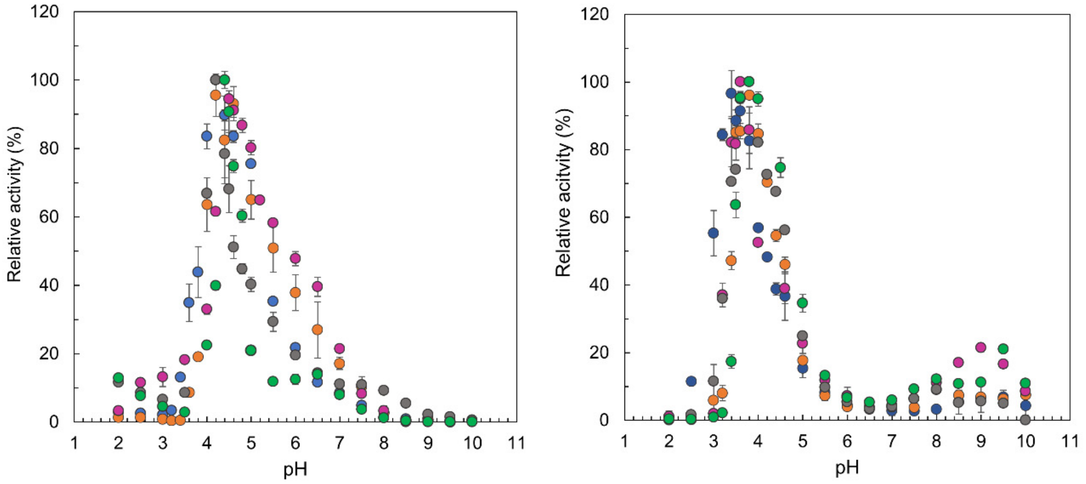

Figure S4. pH profiles of wild-type (blue), and variants 1F9 (orange), 3G5 (grey), 54D6 (pink), and 5G5 (green) for ABTS **(a)** 1 and DMP **(b)**. All assays were performed in the presence of 0.2 mM H_2O_2 using Briton and Robinson buffer (pH range 2 to 10). The relative activity was calculated considering the maximum of activity (at optimal pH) as 100%.

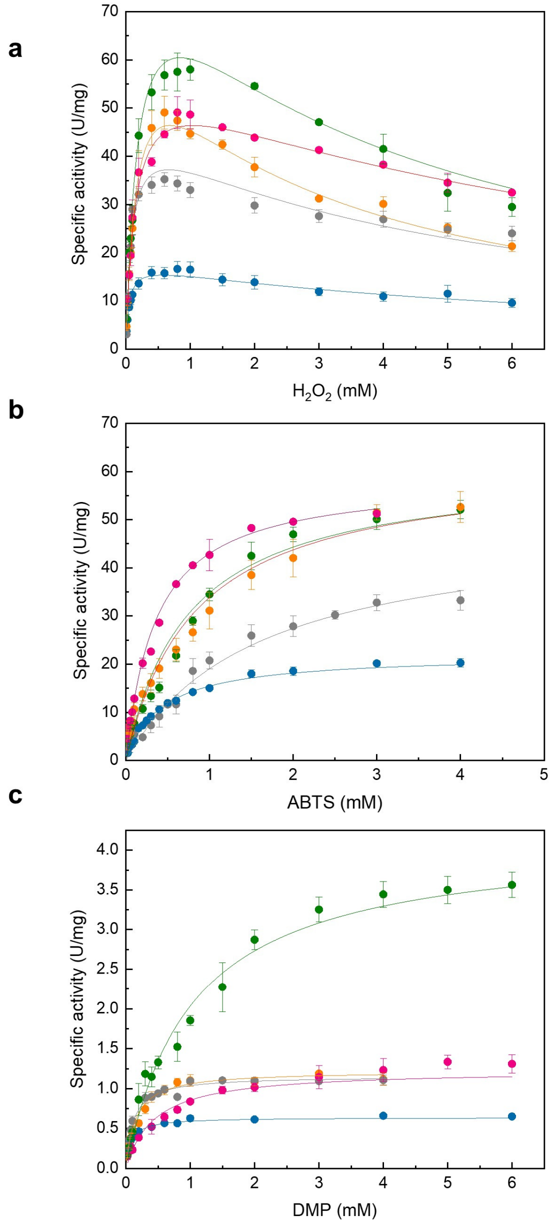

Figure S5. Steady-state apparent kinetic analysis of *Bs*DyP wild-type (blue), and variants 1F9 (orange), 3G5 (grey), 54D6 (pink) and 5G5 (green). Kinetics to obtain catalytic parameters for H_2O_2 were performed using 4 mM ABTS for wild-type, 3G5, 54D6 and 5G5 and 1 mM ABTS for 1F9 **(a)** Kinetics to obtain catalytic parameters for ABTS were performed using 0.2 mM of H_2O_2 for wild-type and 0.6 mM H_2O_2 for 1F9, 3G5, 54D6 and 5G5 **(b)**. Kinetics to obtain catalytic parameters for DMP were performed using 0.2 mM of H_2O_2 for wild-type and 0.6 mM H_2O_2 for 1F9, 3G5, 54D6 and 5G5 **(c)**. All assays were performed at 25ºC, and the reaction mixtures contained 100 mM sodium phosphate buffer pH 4.2 (for H_2O_2 and ABTS assays) and 100 mM sodium acetate buffer pH 3.8 (for DMP assays). Kinetic data was fitted directly using the Michaelis-Menten equation and the substrate inhibition equation ($v = V_{max}[S]/(K_m+[S](1+[S]/K_i))$).

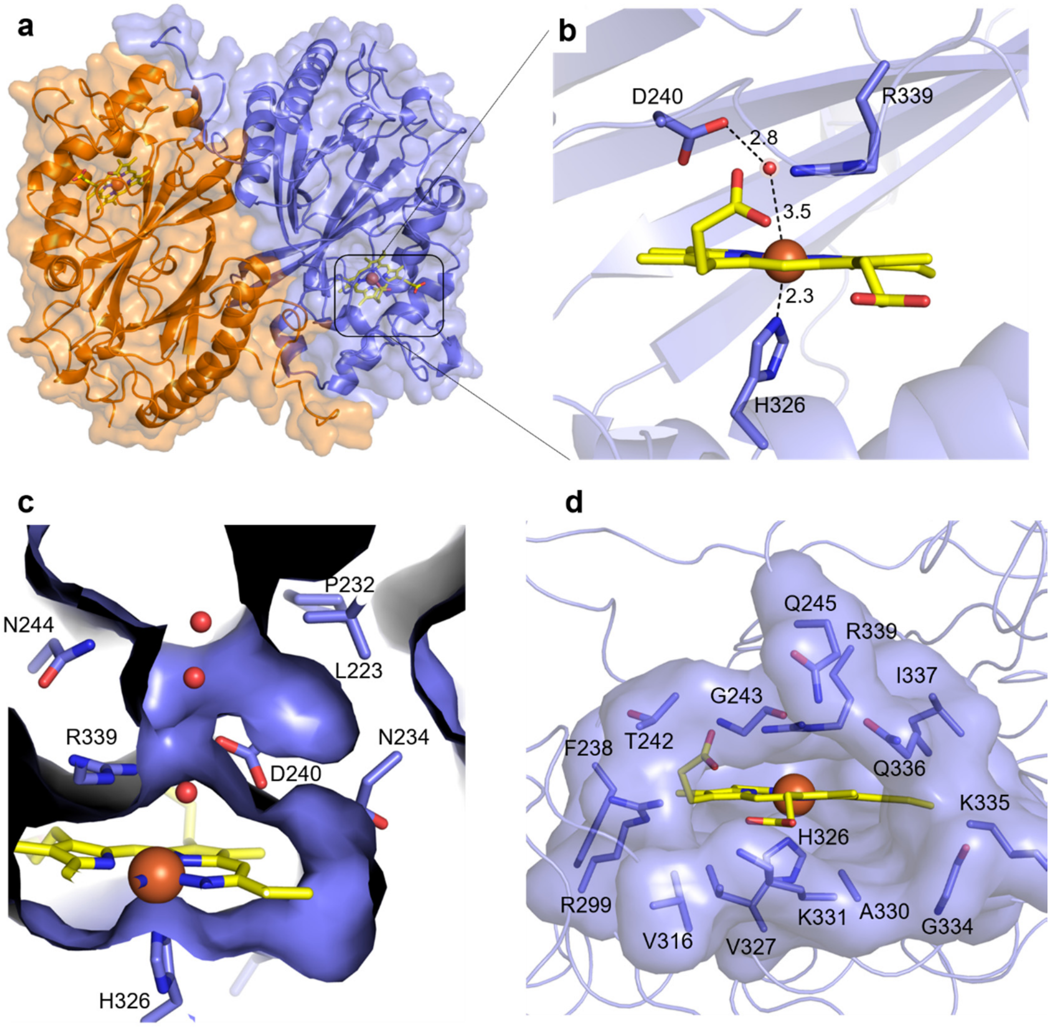

Figure S6. *Bs*DyP wild-type active site and substrate molecular pathways. **(a)** The two crystallographically independent molecules, A and B, which form the asymmetric unit of the structure, are shown as cartoon and transparent molecular surface in blue and orange, respectively. Iron atoms are represented as orange spheres and heme cofactor as sticks with carbon atoms in yellow, nitrogen in blue and oxygen in red. **(b)** Zoomed-in view of the heme active center showing the catalytic distal residues, D240 and R339, and the proximal H326 ligand. The catalytic D240 and R339 side-chains are oriented to the heme iron, with a $D240^{OD1}$-Fe distance of 5.2 Å and $R339^{NH2}$-Fe distance of 4.6 Å. The residues are shown as sticks with carbon; nitrogen and oxygen atoms colored in blue, dark blue and red, respectively. The distance (Å) between D240 and the water molecule and the iron-H326 bond distance are shown in black dashed lines. The water molecule is shown as a red sphere. **(c)** Tunnel defined by a rolling sphere of 1.4 Å connects the external surface with the heme active site. The D240 side-chain is hydrogen bonded to a water molecule and $N234^{ND2}$. The guanidyl group of the R339 is within hydrogen bonding distance to a water molecule, $N244^{OD1}$ and to heme propionate via O1A. An electrostatic interaction between the propionate moieties of the heme and the conserved R339 may help position the heme in the active site. Amino acids lining the tunnel are represented as sticks with carbon, nitrogen and oxygen atoms colored as in (b). **(d)** Cavity defined as a transparent molecular surface allows the heme propionate to access to solvent media. Residues limiting this molecular pathway are shown as sticks with carbon, nitrogen and oxygen atoms colored as in (b).

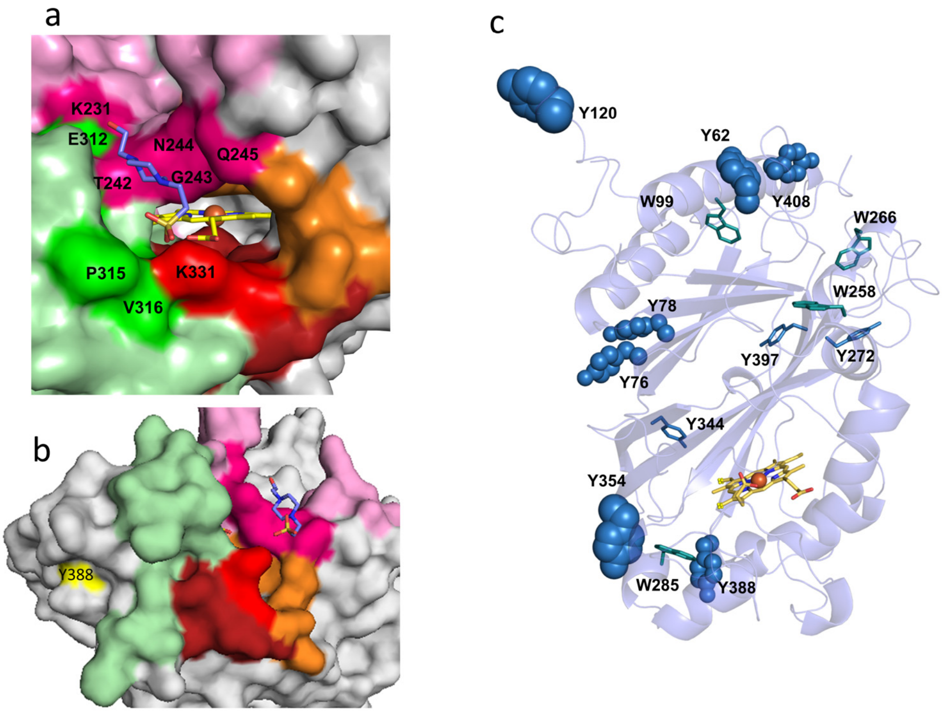

Figure S7. HEPES binding site in *Bs*DyP structure (PDB 7PKX). **(a)** Structural superposition of the BsDyP-HEPES bound (PDB 6KMM) with BsDyP PDB 7PKX reveals that the HEPES molecule interacts with residues from loop 1 (K231, T242, G243, N244 and Q245), loop 2 (E312, P315 and V316) and the small α6-helix (K331). Residues from loop 1 and 2 and small α-helix are colored in pink, light green and red. The HEPES molecule is represented as sticks with carbon atoms in blue, oxygen in red, nitrogen in dark blue, and sulfur in yellow. **(b)** The aromatic residue Y388, colored in yellow, is located in the cavity vicinity within 8 Å of the heme. **(c)** Location of tyrosine (four) and tryptophan (ten) residues in BsDyP (PDB 7PKX). The side chains of residues that are not solvent-exposed are shown as sticks, while the residues exposed to solvent are shown as spheres with radius proportional to their ASA values. Y388, W285 and Y344 are the residues closer to the heme cavity at 8, 9 and 11 Å, respectively, see Table S8. The monomeric unit of BsDyP (PDB 7PKX) is shown in blue.

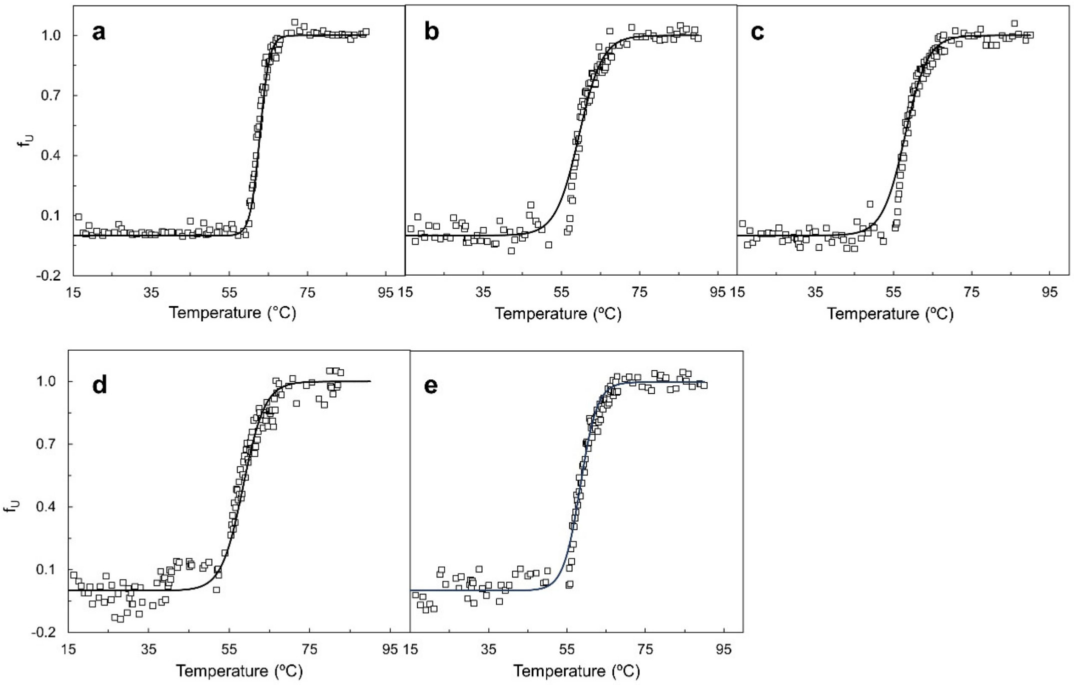

Figure S8. Unfolded fraction (f_U) of *Bs*DyP wild-type **(a)** and variants 1F9 **(b)**, 3G5 **(c)**, 54D6 **(d)** 5G5 **(e)** as a function of the temperature at pH 5.0, determined by fluorescence emission. The solid lines are the fit according to the equation $f_U = \exp^{(-\Delta G°/RT)}/1+\exp^{(-\Delta G°/RT)}$, which assumes the equilibrium N ⇄ U.

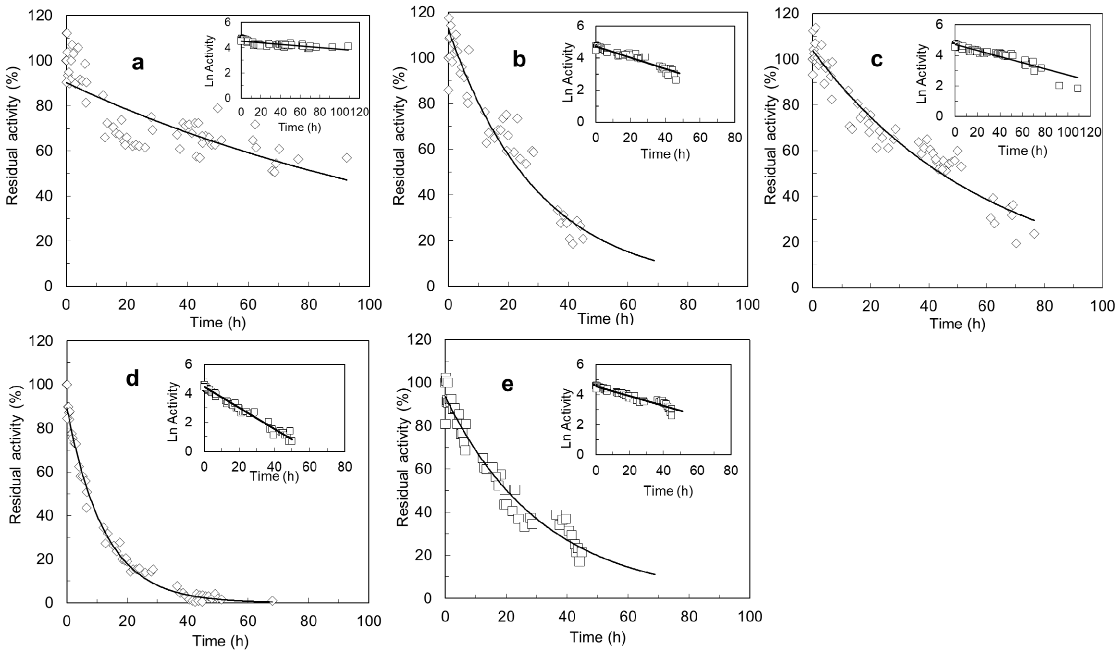

Figure S9. Kinetic stability of *Bs*DyP wild-type and variants. The activity decay at 40ºC was fitted accurately considering an exponential decay with a half-life of 109 ± 1 h (WT) **(a)**, 21 ± 1 h (1F9) **(b),** 27 ± 3 h (3G5) **(c)**, 10 ± 2 h (54D6) **(d)** and 20 ± 4 h (5G5) **(e)**. The inset figures show that the activity decay of *Bs*DyP wild-type and variants can be fitted to a single first-order process, as the logarithm of activity displays an inverse linear relationship with time.
